# Signaling Dependent and Independent Mechanisms in Pemphigus Vulgaris Blister Formation

**DOI:** 10.1371/journal.pone.0050696

**Published:** 2012-12-03

**Authors:** Masataka Saito, Sara N. Stahley, Christopher Y. Caughman, Xuming Mao, Dana K. Tucker, Aimee S. Payne, Masayuki Amagai, Andrew P. Kowalczyk

**Affiliations:** 1 Departments of Cell Biology, Dermatology and the Winship Cancer Institute, Emory University, Atlanta, Georgia, United States of America; 2 Department of Dermatology, University of Pennsylvania, Philadelphia, Pennsylvania, United States of America; 3 Department of Dermatology, Keio University, Tokyo, Japan; NCMLS, Radboud University Nijmegen Medical Center, The Netherlands

## Abstract

Pemphigus vulgaris (PV) is an autoimmune epidermal blistering disease caused by autoantibodies directed against the desmosomal cadherin desmoglein-3 (Dsg3). Significant advances in our understanding of pemphigus pathomechanisms have been derived from the generation of pathogenic monoclonal Dsg3 antibodies. However, conflicting models for pemphigus pathogenicity have arisen from studies using either polyclonal PV patient IgG or monoclonal Dsg3 antibodies. In the present study, the pathogenic mechanisms of polyclonal PV IgG and monoclonal Dsg3 antibodies were directly compared. Polyclonal PV IgG cause extensive clustering and endocytosis of keratinocyte cell surface Dsg3, whereas pathogenic mouse monoclonal antibodies compromise cell-cell adhesion strength without causing these alterations in Dsg3 trafficking. Furthermore, tyrosine kinase or p38 MAPK inhibition prevents loss of keratinocyte adhesion in response to polyclonal PV IgG. In contrast, disruption of adhesion by pathogenic monoclonal antibodies is not prevented by these inhibitors either in vitro or in human skin explants. Our results reveal that the pathogenic activity of polyclonal PV IgG can be attributed to p38 MAPK-dependent clustering and endocytosis of Dsg3, whereas pathogenic monoclonal Dsg3 antibodies can function independently of this pathway. These findings have important implications for understanding pemphigus pathophysiology, and for the design of pemphigus model systems and therapeutic interventions.

## Introduction

Desmosomes are adhesive intercellular junctions which are anchored to the keratin intermediate filament cytoskeleton [1]–[5]. These robust intercellular junctions are prominent in tissues that experience substantial mechanical stress, such as the skin and heart. Desmosomes are composed primarily of desmosomal cadherins, desmogleins and desmocollins, armadillo proteins such as plakoglobin and the plakophilins, and a plakin family member, desmoplakin. Together, these proteins couple calcium-dependent adhesive interactions mediated by the desmosomal cadherins to the intermediate filament cytoskeleton, thereby mechanically coupling adjacent cells [1]–[3]. Although essential for tissue integrity, desmosomes are highly dynamic complexes that are often remodeled during various cellular processes, such as development and wound healing [1], [6].

Pemphigus is a family of potentially fatal autoimmune blistering skin diseases caused by autoantibodies directed against desmosomal cadherins desmoglein 1 (Dsg1) and desmoglein 3 (Dsg3) [7]–[12]. The major forms of pemphigus include pemphigus vulgaris and pemphigus foliaceus. In pemphigus vulgaris (PV), autoantibodies (IgG) are generated against Dsg3, or both Dsg3 and Dsg1. In contrast, pemphigus foliaceus is characterized by antibodies directed against Dsg1 [7], [10]. The histological hallmark of pemphigus is the loss of cell-cell adhesion between epidermal keratinocytes, or acantholysis [7], [10]. Although it is now well-established that PV and PF are caused by antibodies against desmogleins, the precise pathomechanism of pemphigus is not fully understood [11], [13].

A major unresolved question is whether the loss of cell-cell adhesion triggered by pemphigus IgG is caused by direct inhibition of desmoglein cis or trans interactions (steric hindrance), by endocytosis of cell surface Dsg3, by the activation of cellular signaling pathways, or by some combination of these events [11]–[13]. Previous work using atomic force microscopy has shown that IgG from PV patients (PV IgG) can inhibit Dsg3 trans-interactions [14] which mediate cadherin-cadherin binding between adjacent cells [15]. In addition, experimentally generated monoclonal Dsg3 antibodies, Fab fragments of PV patient IgG, and recombinant single chain monovalent fragments of PV patient antibodies have been found to disrupt desmosomal adhesion in various PV model systems [16]–[18]. Pathogenic monoclonal antibodies cloned from PV patients (PV mAbs), as well as experimentally generated antibodies against Dsg3 which cause loss of adhesion, are typically directed against the amino-terminal adhesive interface of Dsg3 [17], [18]. These findings suggest that PV IgG most likely cause loss of adhesion in patients by sterically disrupting Dsg3 adhesive interactions.

Several observations challenge the notion that pemphigus is caused by steric hindrance alone. For example, inhibition of signaling pathways or inhibition of Dsg3 endocytosis can prevent PV IgG-induced loss of adhesion in both cell culture and animal model systems [19]–[26]. Protein kinase C (PKC), RhoA, c-myc, and tyrosine kinase pathways have all been implicated in the signaling pathway leading to loss of adhesion in keratinocytes treated with PV IgG [22]–[27]. A particularly compelling case has been established for p38 MAPK, which has been linked to both Dsg3 endocytosis and the loss of keratinocyte adhesion in response to PV IgG [19], [20], [28]. However, recent studies have shown that p38 alpha MAPK null mice treated with pathogenic Dsg3 monoclonal antibodies exhibit blistering in response to mechanical stress, indicating that p38 MAPK may not be required for these antibodies to disrupt epidermal adhesion in vivo [29].

One explanation that may reconcile these disparate observations is that polyclonal patient IgG disrupts adhesion by a different mechanism than pathogenic mouse monoclonal IgG or PV mAbs cloned from patients. In the present study, we provide evidence that a significant component of the pathogenic activity of PV IgG can be attributed to the polyclonal nature of patient antibodies. We find that the polyclonal aspect of PV patient IgG is responsible for aberrant cell surface clustering and endocytosis of Dsg3, which occur in a p38 MAPK-dependent manner. In contrast, pathogenic monoclonal IgG directed against Dsg3 cause loss of adhesion in a p38 MAPK-independent fashion that is most likely dependent upon the ability of these antibodies to sterically hinder Dsg3 adhesive interactions. These findings have important implications for designing model systems to study pemphigus pathomechanisms and for developing therapies to treat this devastating disease.

## Results

### Polyclonal and Monoclonal Pathogenic Anti-Dsg3 IgG Antibodies Cause Distinct Alterations in Desmosome Morphology

**Figure 1:**
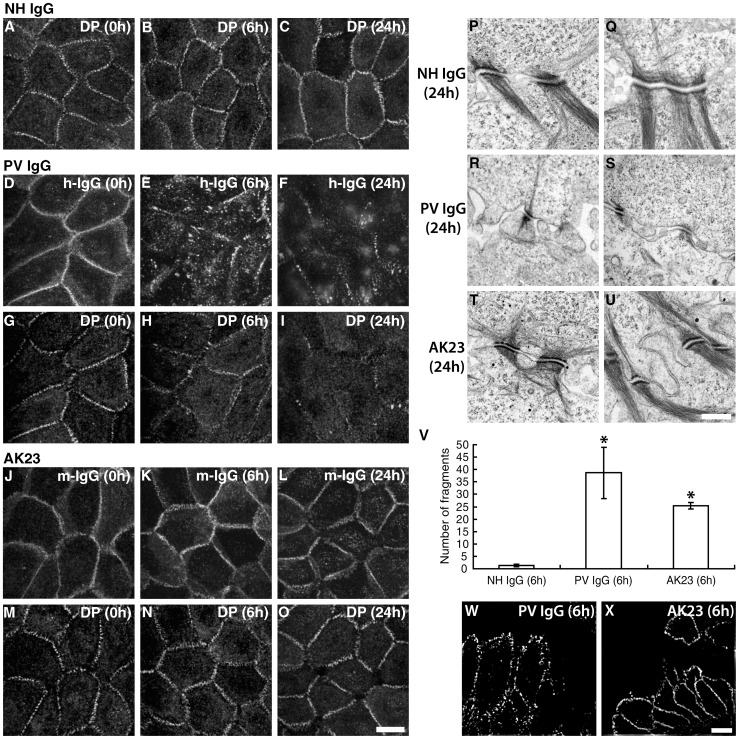
Polyclonal and monoclonal pathogenic Dsg3 antibodies cause distinct morphological changes in desmosomes. (**A–C**) Desmoplakin (DP) localization is unchanged upon addition of NH IgG. (**D–I**) PV IgG bound to cell-cell borders in a linear and punctate pattern in cells incubated at 4°C (time 0). After 6 h at 37°C, PV IgG staining at cell borders became discontinuous (E), and was markedly disrupted by 24 h (F). DP staining also became disorganized by 6 h (H) and deteriorated further by 24 h (I). (**J–O**) In contrast, when cells were treated with AK23, the linear border staining pattern remained largely unchanged even after 24 h (J–L). Similarly, DP staining showed little disruption, although small gaps were occasionally noted between cells after 24 h (M–O). (**P–U**) Desmosomes in cells treated with NH IgG exhibit normal desmosomal morphology (P,Q). Desmosomes in PV IgG-treated cells (R, S) were smaller in size, disorganized and exhibited reduced keratin association. Desmosomes of AK23-treated cells appeared morphologically indistinguishable from control cells (T, U). (**V**) In the dispase-based dissociation assay, keratinocyte sheets treated with NH IgG remained nearly intact, whereas cells treated with either PV IgG or AK23 IgG exhibited extensive fragmentation. (**W–X**) Human skin explants injected with PV IgG (W) or AK23 (X) were analyzed by structured-illumination microscopy (SIM) to further examine PV IgG and AK23 distribution. Images are oriented with dermis down and the basal layer horizontal across the bottom of each panel. Loss of adhesion (acantholysis) is evident by the empty space above the basal layer (a portion of the suprabasal layer is still visible in panel X). See Figure 6 for additional histological analysis of these samples. As observed by SIM, Dsg3 clustering is apparent in skin explants in response to PV IgG (W) but not AK23. *Indicates statistical significance compared to NH IgG (P<0.05). Scale bar for A-O, 10 µm. Scale bar for P-U, 0.5 µm. Scale bar for W-X, 5 µm.

Normal human epidermal keratinocytes were shifted from low calcium conditions to media containing 0.6 mM calcium chloride for 16–18 h and treated either with PV IgG or AK23, a monoclonal mouse IgG antibody directed against the putative adhesive interface near the amino-terminus of Dsg3 [18]. Substantial differences in desmosome protein distribution between PV IgG and AK23-treated cells were observed. Both PV IgG and AK23 initially bound to cell-cell borders in a linear pattern (Fig. 1D, J). After 6 h, however, PV IgG staining at cell borders became discontinuous and was markedly disrupted by 24 h (Fig. 1E, F). In parallel with these changes in PV IgG localization, desmoplakin (DP) staining also became disorganized by 6 h and deteriorated further by 24 h (Fig. 1G, H, I). In contrast, when cells were treated with AK23, the linear border staining pattern at 0 h remained largely unchanged even after 24 h (Fig. 1J, K, L). Similarly, DP staining showed little disruption, although small gaps were occasionally noted between cells after 24 h (Fig. 1M, N, O). Similar results were obtained using a high dose treatment of AK23 (Fig. S1) and using both pathogenic and non-pathogenic single chain monvalent mAbs (scFv) cloned from human PV patients [17] (Fig. S2). Electron microscopy was used to evaluate ultrastructural changes in desmosome morphology. In PV IgG-treated cultured keratinocytes, desmosomes were smaller in size, disorganized and exhibited reduced keratin association compared to control cells treated with normal human IgG (NH IgG) (Fig. 1P, Q, R, S). Remarkably, desmosomes of AK23-treated cells appeared morphologically indistinguishable from control cells treated with NH IgG (Fig. 1T, U). To assess the functional status of desmosomal adhesion in these cells, keratinocyte sheets were incubated with antibodies, removed from the substrate using the enzyme dispase and then subjected to mechanical stress as described previously [30]. Keratinocyte sheets treated with NH IgG remained intact, whereas cells treated with either PV IgG or AK23 IgG exhibited extensive fragmentation (Fig. 1V). Thus, although PV IgG and AK23 both induced loss of cell adhesion strength, substantial changes in desmosomal protein distribution and ultrastructual features of desmosomes were caused by PV IgG, but not AK23. To further examine how PV IgG and AK23 distribution differ in human epidermis, antibodies were injected into human skin explant cultures and then examined after 16 h using super-resolution immunofluorescence microscopy. Both PV IgG and AK23 caused loss of epidermal cell adhesion as evidenced by separation of basal epidermal cells from suprabasal cell layers (Fig. 1 W, X). Similar to cultured cells, PV IgG were distributed in a more clustered and punctate appearance in basal keratinocytes compared to AK23, which remained in a more diffuse pattern along keratinocyte membranes. These results suggest that the pathogenic mechanism causing loss of cell-cell adhesion differs between the polyclonal and monoclonal antibodies.

### Polyclonal IgG Antibodies Cause Clustering and Endocytosis of Dsg3

**Figure 2:**
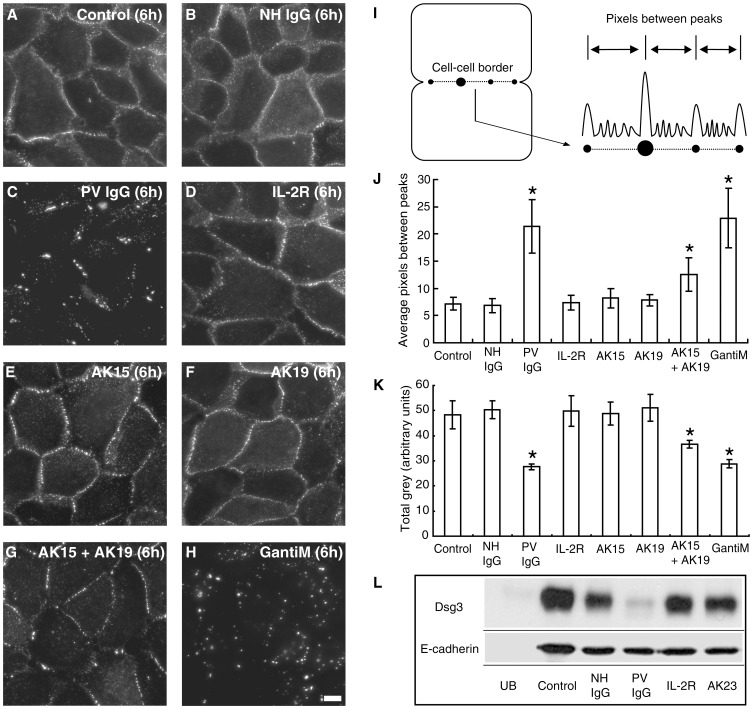
PV IgG causes clustering and loss of surface Dsg3. (**A–H**) Cell surface Dsg3 was pulse-labeled with biotinylated AK23 and detected using streptavidin-conjugated Alexa Fluor 555. AK23-bound Dsg3 (AK23-Dsg3) was found primarily at cell-cell junctions on the cell surface after 6 h with either no antibody (control) (A) or with NH IgG (B). Addition of PV IgG caused a redistribution of surface AK23-Dsg3 into highly concentrated regions of staining along cell borders (C). Addition of an irrelevant monoclonal antibody against the IL2-receptor caused no change in AK23-Dsg3 distribution (D). When AK15 or AK19 were added, AK23-Dsg3 border staining remained similar to controls (E,F). However, addition of both AK15 and AK19 together resulted in a highly punctate pattern of AK23-Dsg3 at borders (G). Similarly, addition of goat anti-mouse IgG clustered AK23-Dsg3 (H). (**I**) Procedure for measuring distance between peak fluorescent intensities. (**J**) Quantitative assessment revealed that the distance between AK23-Dsg3 peak fluorescence intensity increased in cells treated with PV IgG, the AK15/19 combination, or goat anti-mouse IgG. (**K**) Total surface fluorescence intensity measurements (Total grey) demonstrated that PV IgG, the AK15/19 mixture, as well as goat anti-mouse IgG reduced Dsg3 cell surface levels. (**L**) In cell surface biotinylation analysis, PV IgG reduced Dsg3 surface levels, while cell surface Dsg3 remained relatively stable even after 24 h in cells treated with NH IgG or pathogenic doses of AK23. UB indicates unbiotinylated sample. Control reflects total surface levels at 0 hr. *Indicates statistical significance compared to control (P<0.05). Scale bar in A-H, 10 µm.

To define the mechanism by which PV IgG disrupt desmosomes, a pulse-chase study was performed in which cell surface Dsg3 was labeled with biotinylated AK23 (AK23-biotin) at a non-pathogenic dose [31]. Cells were then exposed to various antibodies or antibody combinations, and then fixed with paraformaldehyde without permeabilization. Cell surface AK23-Dsg3 complexes were then detected using streptavidin-conjugated Alexa Fluor 555. Consistent with the results shown in Figure 1, in the absence of additional antibodies or in the presence of NH IgG, the AK23-Dsg3 complex was found to remain primarily at cell-cell junctions on the cell surface after 6 h incubation (Fig. 2A–B). However, addition of PV IgG caused a redistribution of the cell surface AK23-Dsg3 complex into highly concentrated regions of staining along cell borders, indicating that PV IgG triggered clustering of Dsg3 molecules (Fig. 2C). To test if this change was due to the polyclonal nature of PV IgG, two additional mouse monoclonal IgG antibodies against Dsg3 (AK15 and AK19) were used to simulate the polyclonal mixture of anti-Dsg3 IgG present in PV patient IgG. When either AK15 or AK19 alone was added, AK23-Dsg3 border staining remained similar to control, although the presence of some small puncta were noted (Fig. 2E, F). However, addition of both AK15 and AK19 together resulted in a highly punctate pattern of AK23-Dsg3 at cell-cell borders (Fig. 2G). To confirm that these changes were due to clustering, goat anti-mouse IgG polyclonal antibodies were used to crosslink cell surface AK23-Dsg3 complexes. As expected, significant clustering of the AK23-Dsg3 complex was observed after addition of goat anti-mouse IgG (Fig. 2H). Quantitative assessment (Fig. 2I) revealed that the distance between clusters of AK23-Dsg3 increased in cells treated with PV IgG (Fig. 2J). Similar quantitative changes were observed in cells treated with the combination of AK15 and AK19, or with goat anti mouse IgG. To verify that the AK23-biotin labeling did not cause any change in Dsg3 clustering over the time course of the experiment, cells that were fixed prior to AK23 addition were compared to cells that were pulse-labeled with AK23 and then incubated for 6 h at 37°C. Even after a 6 h chase period, the distance between AK23 peak fluorescence intensity along borders did not change when compared to staining of fixed cells (Fig. S3), demonstrating that the AK23 labeling procedure did not induce changes in Dsg3 distribution. Furthermore, analysis of total cell surface fluorescence intensity measurements demonstrated that PV IgG, the AK15 and 19 mixture, as well as goat anti mouse IgG all reduced cell surface levels of Dsg3 (Fig. 2K). To assess Dsg3 levels biochemically, keratinocyte cell surface proteins were labeled with biotin before treatment with various IgG combinations. After 24 h, cells were harvested, remaining biotinylated cell surface proteins captured using streptavidin resin, and captured proteins subjected to western blotting analysis to monitor relative amounts of cell surface Dsg3. In cells treated with PV IgG, surface levels of Dsg3 were dramatically reduced, consistent with previous studies from our lab [26], [30], [31] and others [28], [29], [32], [33] showing that PV IgG cause Dsg3 endocytosis (Fig. 2L). In contrast, cell surface Dsg3 remained relatively stable even after 24 h in cells treated with NH IgG. Interestingly, treatment with pathogenic doses of AK23 did not cause down-regulation of cell surface Dsg3 levels. Together, these data suggest that clustering of Dsg3 molecules on the cell surface due to crosslinking by polyclonal antibodies triggers Dsg3 endocytosis and depletion of Dsg3 cell surface levels. In contrast, pathogenic doses of AK23 that disrupt keratinocyte cell-cell adhesion do not cause significant clustering or endocytosis of cell surface Dsg3.

### Loss of Adhesion Does not Cause Dsg3 Endocytosis

**Figure 3:**
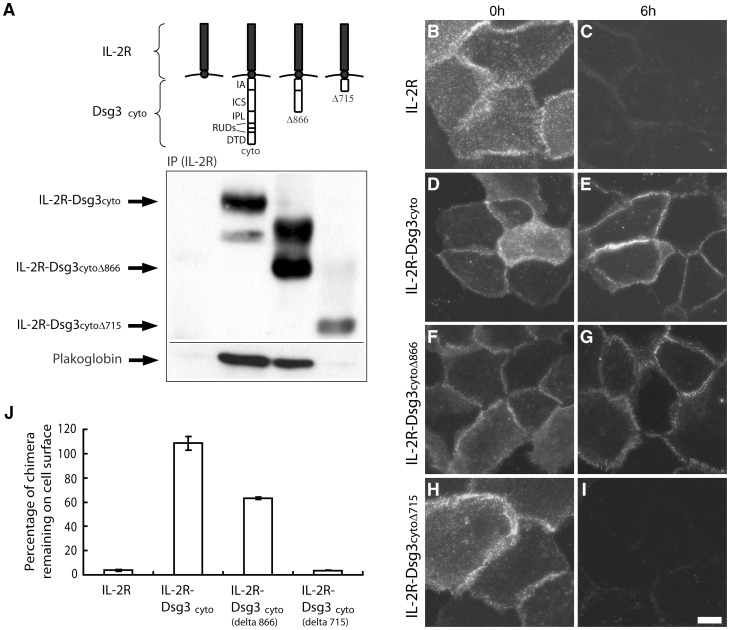
Loss of adhesion does not cause Dsg3 endocytosis. (**A**) The Dsg3 cytoplasmic domain was fused to the non-adhesive extracellular domain of the IL-2 receptor (IL2R) alpha chain. Carboxyl terminal deletion mutants were also generated to remove various Dsg3 cytoplasmic domains as illustrated. Immunoprecipitation and western blot analysis verified expression of each construct and demonstrated that deletion of the ICS domain abolished plakoglobin binding. (**B–I**) Antibody labeling of cells incubated at 4°C demonstrated that each of the chimeric IL2R polypeptides were delivered to the cell surface (B,D,F,H). After 6 h at 37°C, cell surface levels of IL-2R were significantly diminished (C). In contrast, the non-adhesive IL-2R-Dsg3_cyto_ was stable at the cell surface over the 6 hr time course (E). Deletion of the DTD and RUD domains resulted in more rapid loss of surface pools of the Dsg3 chimera (F–G, J), and further removal of the IPL and ICS domains enhanced this effect (H–I, J). (**J**) Quantitative analysis of cell surface levels remaining at 6 hr normalized to chimera levels at 0 h. IA, intracellular anchor; ICS, intracellular cadherin-like sequence; IPL, intracellular proline-rich linker; RUD, repeat unit domain; DTD, desmoglein terminal domain.

The observation that AK23 caused loss of cell adhesion in the dispase-based dissociation assay but no change in cell surface levels of Dsg3 suggested that loss of adhesion alone does not cause Dsg3 endocytosis. To determine if non-adhesive Dsg3 polypeptides are stable on the cell surface, we fused the Dsg3 cytoplasmic domain to the non-adhesive Interleukin 2 receptor (IL-2R) alpha chain extracellular domain (Fig. 3A). In previous studies, we have shown that addition of the IL-2R extracellular domain to classical cadherins results in dramatically increased rates of endocytosis compared to the IL-2R extracellular domain without a cadherin tail [34], [35]. Therefore, keratinocytes were infected with adenovirus expressing the IL-2R or the IL-2R-Dsg3_cyto_ chimera. Cell surface pools of the proteins were labeled with biotinylated anti-IL-2R antibody, incubated for 6 h, fixed and stained with Alexa fluor 555-conjugated streptavidin to detect only the cell surface IL-2R or the IL-2R-Dsg3_cyto_ chimera. As shown in Figure 3 (B, D), IL-2R and IL-2R-Dsg3_cyto_ both can be seen on the cell surface (0 h). After 6 h, surface pools of IL-2R were significantly diminished (Fig. 3C, J). In contrast, the non-adhesive IL-2R-Dsg3_cyto_ was remarkably stable (Fig. 3E, J). These results suggested that cytoplasmic interactions rather than adhesive interactions regulate Dsg3 endocytosis. To test this possibility, a series of deletion constructs of the IL-2R-Dsg3_cyto_ chimera were generated in which the DTD (desmoglein terminal domain) and RUD (repeating unit domain) domains (IL-2R-Dsg3_cytoΔ866_) or the RUD, IPL (intracellular proline-rich linker) and ICS (intracellular cadherin-like sequence) domains (IL-2R-Dsg3_cytoΔ715_) were removed (Fig. 3A). Immunoprecipitation and western blot analysis verified expression of each construct and demonstrated that deletion of the ICS domain abolished plakoglobin binding (Fig. 3A). Interestingly, deletion of the DTD and RUD domains resulted in more rapid loss of surface pools of the chimera, and further removal of the plakoglobin binding domain (ICS) enhanced this effect (Fig. 3G, I, J). Taken together, these data indicate that a lack of Dsg3 transinteraction does not cause internalization, and that the Dsg3 tail prevents internalization.

### Genistein Prevents Loss of Adhesion Caused by PV IgG, but not AK23

**Figure 4:**
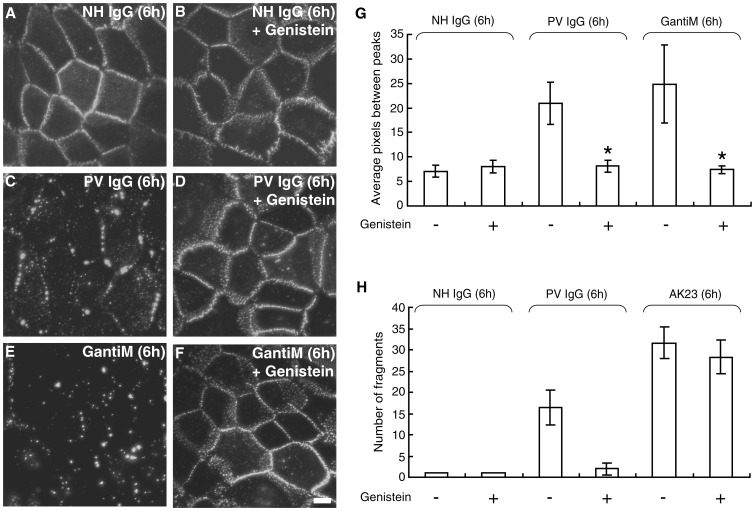
Genistein prevents PV IgG-induced clustering and loss of adhesion. **A–F**) Cell surface Dsg3 was labeled with biotin-conjugated AK23 monoclonal antibody followed by addition of NH IgG (A,B) PV IgG (C,D) or goat anti-mouse IgG (E,F) in the absence (A,C,E) or presence (B,D,F) of the pan-tyrosine kinase inhibitor genistein. Treatment of cells with genistein prevented PV IgG- and goat anti-mouse IgG induced clustering of the AK23-Dsg3 complex (D,F). (**G**) Quantitative assessment of clustering. (**H**) In the dispase-based cell dissociation assay, genistein potently prevented loss of adhesion (monolayer fragmentation) in keratinocytes treated with PV IgG. In contrast, genistein treatment was unable to prevent loss of adhesion induced by AK23. *Indicates statistically significant differences with genistein treatment (P<0.05). Scale bar, 10 µm.

We have previously shown that a pan-tyrosine kinase inhibitor, genistein, inhibits both endocytosis of Dsg3 and loss of keratinocyte adhesion in response to PV patient IgG [26]. Since we observed a correlation between clustering and endocytosis of Dsg3, we tested whether genistein affects clustering of Dsg3 caused by PV IgG. Similar to the results shown in Figure 2, PV IgG, but not NH IgG caused extensive clustering and punctate border staining of cell surface Dsg3 (Fig. 4A, C, G). Treatment of cells with genistein prevented PV IgG-induced clustering of Dsg3 (Fig. 4D, G). To confirm that genistein inhibits Dsg3 clustering, we next tested if genistein could also prevent clustering of AK23-Dsg3 complexes by the addition of goat anti mouse IgG. Importantly, genistein also inhibited clustering of AK23-Dsg3 complexes caused by the addition of goat anti-mouse secondary antibodies (Fig. 4E, F, G).

The observation that PV IgG causes clustering which can be inhibited by genistein, along with the finding that AK23 disrupts adhesion strength without causing clustering and endocytosis, suggested that genistein may protect keratinocyte adhesion from PV IgG but not AK23. To test this possibility, keratinocyte cell-cell adhesive strength was examined using the dispase dissociation assay. As previously shown [26], genistein potently prevents loss of adhesion in keratinocytes treated with PV IgG (Fig. 4H) [26]. In contrast, genistein failed to prevent loss of adhesion in cells incubated with AK23. Collectively, these data indicate that clustering and endocytosis of Dsg3 contribute significantly to the pathogenic activity of PV IgG but not AK23.

### p38 MAPK Activity is Required for Dsg3 Clustering and Endocytosis in Response to PV IgG

**Figure 5:**
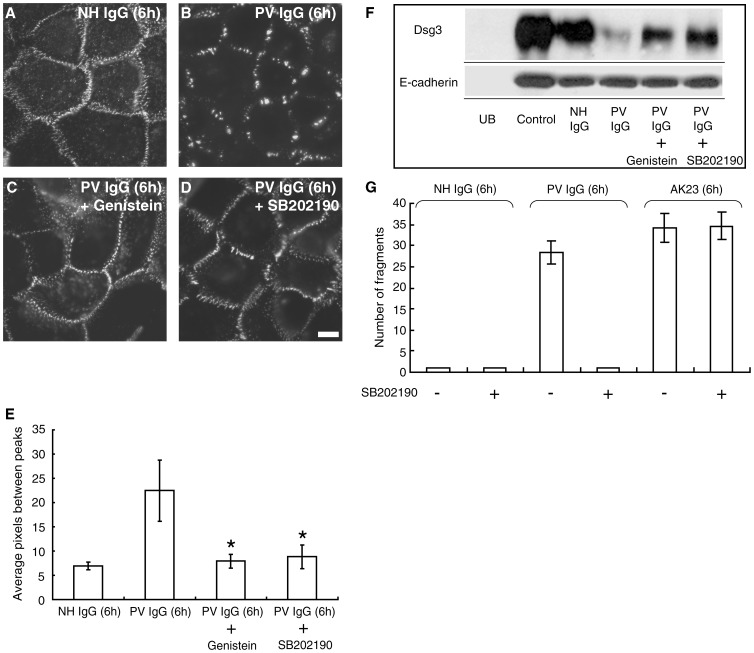
p38MAPK inhibition prevents PV IgG-induced clustering and loss of adhesion. (**A–D**) Cell surface Dsg3 was labeled with biotin-conjugated AK23 monoclonal antibody followed by addition of NH IgG (A) or PV IgG (B-D). Cells in panel C were pretreated with genistein and cells in panel D were pretreated with the p38MAPK inhibitor SB202190 prior to addition of PV IgG. (**E**) Quantitative assessment of clustering. (**F**) Cell surface biotinylation analysis. Genistein and SB202190 prevented loss of cell surface Dsg3 caused by PV IgG. UB represents unbiotinylated sample and control indicates total cell surface Dsg3 at time 0 hr. (**G**) In the dispase-based cell dissociation assay, SB202190 potently prevented loss of adhesion (monolayer fragmentation) in keratinocytes treated with PV IgG but not AK23. *Indicates statistical significance compared to PV IgG (P<0.05). Scale bar, 10 µm.

**Figure 6:**
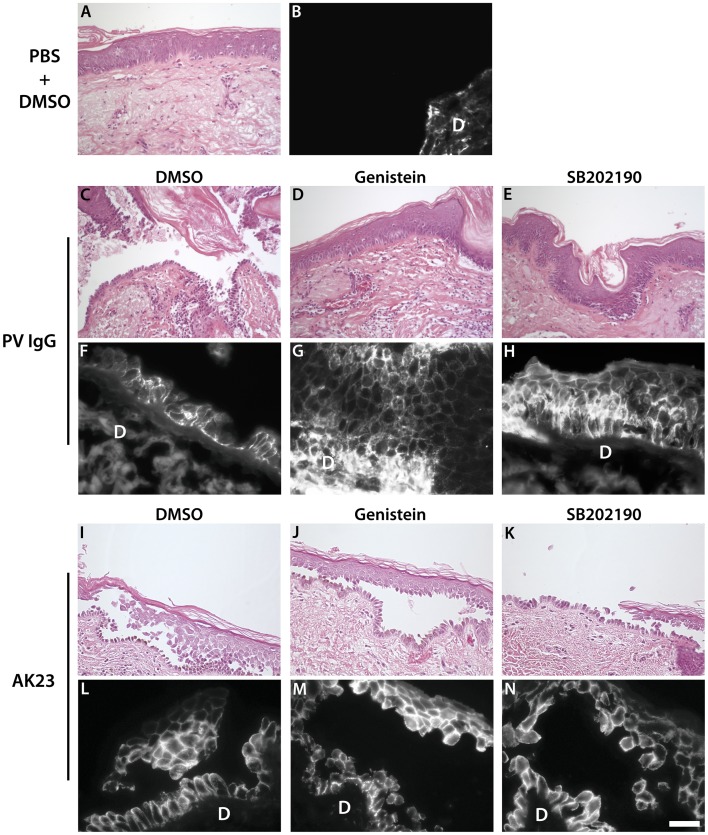
Genistein and SB202190 prevent adhesion loss in skin treated with PV IgG but not AK23. (**A–B**) Human skin explant injected with PBS following a pre-treatment with DMSO. Epidermal morphology is normal (A) with no detectable bound IgG in the epidermis (B). (**C**–**H**) Human skin explants injected with PV IgG following a pre-treatment with DMSO (C,F), genistein (D,G) or SB202190 (E,H). PV IgG caused epidermal acantholysis (loss of cell-cell adhesion) typical of that observed in PV patients (C). Genistein and the p38MAPK inhibitor (SB202190) both prevented acantholysis in skin sections injected with PV patient IgG (D and E respectively). (**I–N**) Human skin explants injected with AK23 following a pre-treatment with DMSO (I,L), genistein (J,M) or SB202190 (K,N). AK23 also caused epidermal acantholysis typical of PV (I). Neither genistein nor the p38MAPK inhibitor (SB202190) prevented acantholysis in skin sections injected with AK23 (J and K respectively). PV IgG (F,G,H) and AK23 (L,M,N) deposition in the basal and immediate suprabasal layers of the epidermis was verified by direct immunofluorescence using goat anti-human and goat anti-mouse antibodies respectively. D = dermis. Scale bar, 20 µm.

The above results suggest that inhibition of signaling pathways that regulate desmosome function might be effective against PV IgG but not monoclonal pathogenic IgG. Recently, it was shown that p38 MAPK signaling and Dsg3 internalization are linked events in pemphigus acantholysis [28]. Therefore, we tested if a well characterized p38 MAPK inhibitor also exhibited differential efficacy against polyclonal vs. monoclonal Dsg3 pathogenic antibodies. Interestingly, in cells treated with the p38 MAPK inhibitor SB202190, PV IgG failed to cause either clustering or loss of cell surface Dsg3, results which were almost identical to those obtained using genistein (Fig. 5A, B, C, D, F). In addition, SB202190 prevented dissociation of PV IgG-treated keratinocyte sheets (Fig. 5G). However, SB202190 was unable to prevent fragmentation caused by AK23 in the dissociation assay, indicating that loss of adhesion induced by AK23 does not require p38 MAPK signaling. To further verify these results, excised human skin was injected with PV IgG or AK23 in the presence or absence of genistein or SB202190 (Fig. 6). Similar to the results obtained in vitro, genistein and SB202190 blocked acantholysis in human skin caused by PV IgG (Fig. 6C, D, E, F, G, H) but not by AK23 (Fig. 6I, J, K, M, N). Together, these data further indicate that the primary mechanism causing loss of cell adhesion is distinct between polyclonal PV patient IgG and monoclonal Dsg3 antibodies.

### EC1 Domain Antibodies are not Required for Dsg3 Clustering

**Figure 7:**
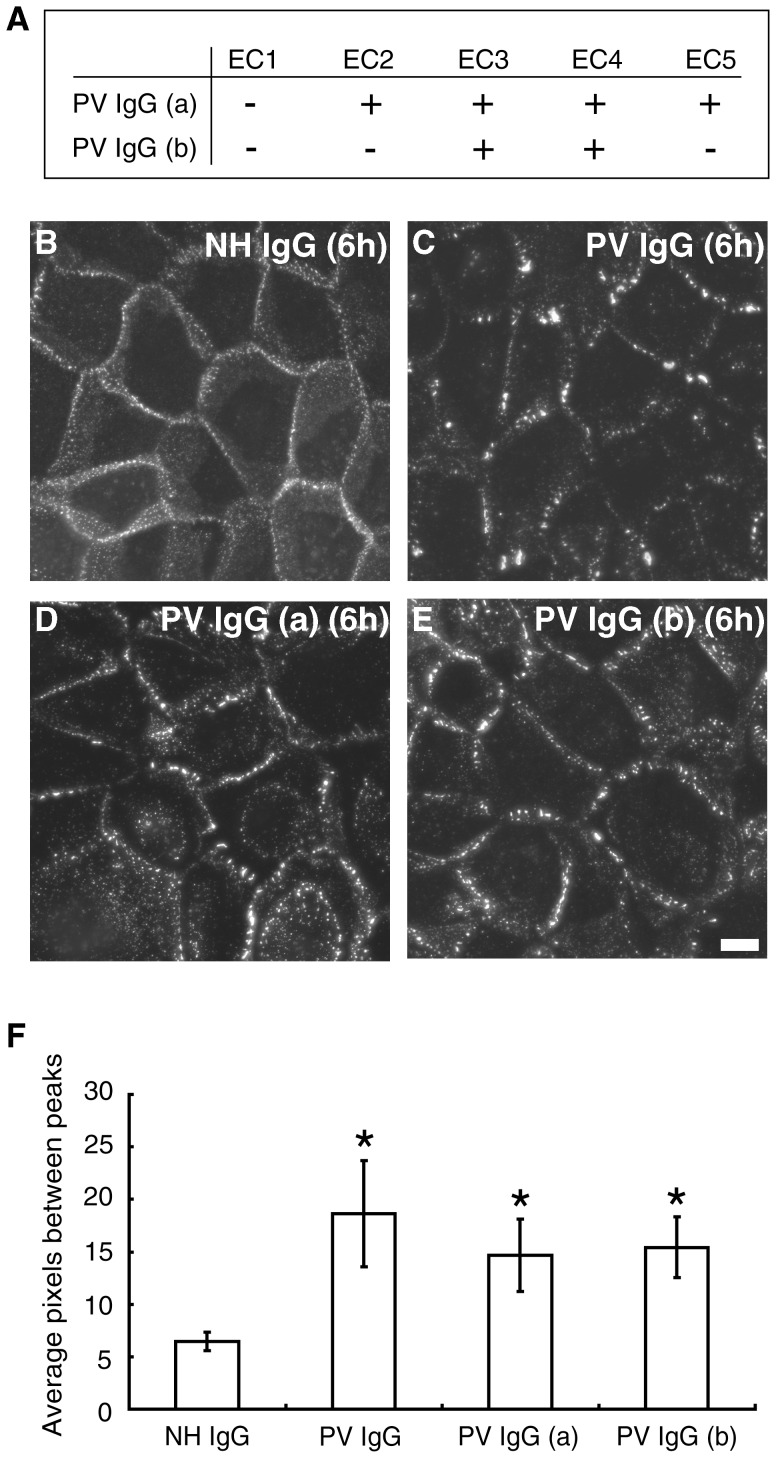
PV IgG lacking antibodies directed against the Dsg3 EC1 domain cause Dsg3 clustering. (**A**) Two PV patient samples, PV IgGa (patient #3270) and PV IgGb (patient #7860) were identified in which epitope mapping revealed the absence of antibodies directed against the N-terminal extracellular domain (EC1) of Dsg3. (**B–E**) Cell surface Dsg3 was monitored using biotinylated AK23. NH IgG caused no change in the distribution of cell surface Dsg3 (B) whereas a typical PV IgG containing EC1 domain antibodies caused Dsg3 clustering (C). Both PV IgG(a) and PV IgG(b) caused Dsg3 clustering (D,E). (**F**) Quantitative assessment of clustering. *Indicates statistical significance compared to NH IgG (P<0.05). Scale bar, 10 µm.

A prediction derived from the results above is that antibodies directed against the Dsg3 adhesive interface would not be required for either loss of adhesion in patients or for Dsg3 clustering in vitro. To test this possibility, PV patient samples lacking antibodies against the N-terminal extracellular (EC1) domain of Dsg3 were evaluated (Representative clinical and histological features of such patients are shown in Fig. S4). Two patient samples, PV IgG (a) and PV IgG (b), were found to contain antibodies against the EC2-5 and EC3-4 domains, respectively, but not against the EC1 domain, as determined by epitope mapping (Fig. 7A). Both PV IgG (a) and (b) caused Dsg3 clustering at cell-cell borders qualitatively and quantitatively similar to more typical PV IgG samples which contain EC1 domain antibodies (Fig. 7B, C, D, E, F). Furthermore, clustering induced by these antibodies was sensitive to p38 MAPK inhibition (Fig. S5). These data indicate that EC1 domain antibodies are not required for active disease in patients and suggest that Dsg3 clustering and endocytosis of Dsg3 are key activities of pathogenic patient antibodies.

## Discussion

The results of this study demonstrate that polyclonal PV patient IgG cause clustering of cell surface Dsg3, resulting in Dsg3 endocytosis and alterations in desmosome morphology. These changes were abrogated by the tyrosine kinase inhibitor genistein and by inhibition of the p38 MAPK pathway, both of which prevented loss of adhesion in PV IgG-treated keratinocytes. In contrast, pathogenic monoclonal antibodies, including AK23 and PV mAbs isolated from patients, failed to cause alterations in desmosomal protein organization. Furthermore, the pathogenic activity of AK23 was resistant to both tyrosine kinase and p38 MAPK inhibition. These results reveal signaling dependent and independent mechanisms for loss of adhesion in response to pathogenic Dsg3 antibodies. These findings provide new insights into how pemphigus patient IgG cause loss of keratinocyte adhesion, and have implications for designing model systems to evaluate therapeutic approaches for treating pemphigus.

It is now well-understood that the acantholysis in pemphigus is a consequence of desmosome disruption caused by autoantibodies against desmosomal cadherins [7]–[11]. Nevertheless, detailed molecular mechanisms leading to desmosomal disruption subsequent to antibody binding are still controversial [11], [13]. Initially, autoantibodies were thought to cause direct inhibition of desmosomal cadherin trans-interactions, a hypothesis supported by studies showing that dominant epitopes recognized by pemphigus antibodies map predominantly to the amino-terminal domain of desmogleins [36]–[39]. Furthermore, a mouse monoclonal antibody directed against the amino-terminal adhesive interface of Dsg3 induces the pemphigus phenotype in mice [18], and a study using atomic force microscopy revealed that PV IgG can directly inhibit Dsg3-mediated trans-interactions [14]. Nonetheless, signaling pathways involving p38 MAPK, RhoA, PKC, c-myc, and plakoglobin have been shown to play a role in PV pathogenesis [19]–[25], [40], [41]. Interestingly, p38 MAPK activity has been linked to internalization and depletion of Dsg3 in PV [19], [20], [28]. Previous studies from our lab and others have also shown that binding of autoantibodies to Dsg3 causes internalization, leading to depletion of desmosomal Dsg3, and consequently desmosome disassembly and loss of adhesion [26], [30], [33], [42]–[45]. On the other hand, a recent study using PV mAbs indicated that p38 MAPK activation is not required for loss of adhesion, but may function downstream of antibody binding to augment Dsg3 endocytosis [29]. Thus, studies have yielded conflicting results that place loss of adhesion either upstream or downstream of signaling pathways that regulate desmosome disassembly in response to pathogenic pemphigus antibodies.

Previous pemphigus research has relied predominantly on PV IgG, which are isolated from patient sera and therefore represent a polyclonal mixture of IgG. However, mouse monoclonal anti-Dsg3 antibodies, such as AK23, or human monoclonal anti-Dsg3 antibodies are now being used extensively to model PV pathogenesis [17], [18], [29], [32], [46], [47]. The advantage of these antibodies is their well-characterized specificity for Dsg3, and the greater reproducibility associated with using monoclonal reagents rather than patient IgG, which can vary between individuals and over the course of disease progression [36], [48]. Mapping of the epitopes recognized by pathogenic IgG from patients strongly supports the notion that some fraction of patient IgG recognizes the Dsg3 adhesive interface, suggesting that these antibodies function by sterically preventing Dsg3 cis- or trans-interactions [36], [37]. However, the remaining IgG that recognize other Dsg3 epitopes outside of the adhesive interface may also contribute to pathogenicity. This idea is further supported by the occurrence of PV sera which lack antibodies against the N-terminal adhesive region of Dsg3 (Fig. 7, Fig. S4) [36]. Previous studies have shown that combined mouse monoclonal anti-Dsg3 antibodies, which mimic the polyclonal nature of PV IgG, had synergistic pathogenic effects on PV blister formation [46]. These findings are consistent with the results presented here showing that the polyclonal nature of patient IgG may alter desmosome function through mechanisms that differ significantly from monoclonal reagents.

The results presented here demonstrate that AK23 as well as PV mAbs induce loss of cell adhesion without causing obvious redistribution of desmosomal proteins, or depletion of cell surface pools of Dsg3. Ultrastructural analysis confirmed that AK23 used at pathogenic doses caused little or no change in desmosome structure (Fig. 1T, U). Presumably, desmosome splitting would occur upon introduction of mechanical stresses seen in vivo [49], [50]. Consistent with this interpretation, previous studies of mouse epidermis exposed to AK23 revealed desmosome splitting, but very little disorganization of desmosomes [51]. Electron microscopy analysis of PV IgG treated keratinocytes suggests that desmosomes disassemble at the cell surface (Fig. 1R, S) rather than being internalized whole, although we cannot rule out the possibility that internalization of half or whole desmosomes may occur in some cells.

A detailed analysis of Dsg3 cell surface distribution revealed that polyclonal PV IgG, but not monoclonal antibodies, cause cell surface clustering of Dsg3 and desmosome disassembly (Fig. 2). Since PV IgG contain divalent IgG antibodies against multiple different epitopes of Dsg3, polyclonal PV IgG has the ability to crosslink Dsg3 molecules on the cell surface. The results presented here are consistent with the clustered appearance of IgG observed in pemphigus patient biopsies [52]. The ability of patient antibodies to cluster Dsg3 appears to be critical for causing Dsg3 endocytosis. Similarly, we find that a mixture of AK antibodies caused both clustering and loss of cell surface Dsg3 (Fig. 2), consistent with previous work showing that a combination of Dsg3 monoclonal antibodies was more effective at causing depletion of steady state Dsg3 than single monoclonal antibodies [33]. Previous studies have found that pathogenic monoclonal antibodies can cause Dsg3 down-regulation [32], [33]. In these previous experiments, keratinocytes were exposed to pathogenic antibodies upon switching cells from low to high calcium medium to trigger desmosome assembly. This approach is in contrast to the present study where antibodies were added to keratinocytes after desmosomes were fully assembled. Thus, Dsg3 may be highly susceptible to antibody-mediated internalization when being trafficked to desmosomes during the assembly process. Nonetheless, the results presented here clearly demonstrate that polyclonal IgG mixtures are significantly more effective at triggering Dsg3 endocytosis than monoclonal antibodies. Furthermore, we found that the non-adhesive Dsg3 chimeric polypeptide IL-2R-Dsg3_cyto_ was stable on the cell surface for 6 hours (Fig. 3). These data suggest that the loss of Dsg3 trans-interactions does not cause Dsg3 endocytosis, although we cannot rule out the possibility that antibody targeting of different Dsg3 epitopes could be differentially effective at triggering Dsg3 internalization. Nonetheless, our results suggest that the primary mechanism by which PV IgG cause loss of steady state Dsg3 levels is through multivalent clustering, leading to Dsg3 endocytosis.

Clustering and endocytosis of Dsg3 in response to polyclonal IgG was prevented by treating with either genistein or the p38 MAPK inhibitor SB202190. In addition, PV IgG-induced fragmentation in the dissociation assay in vitro (Fig. 5) and acantholysis in human skin samples (Fig. 6) was almost completely blocked by these treatments. By striking contrast, neither genistein nor SB202190 protected against the loss of adhesion caused by AK23. The primary pathogenic activity of AK23 can most likely be attributed to steric hindrance. However, it has also been shown that PV IgG caused direct inhibition of Dsg3 trans-interaction as assessed by atomic force microscopy [14]. Thus, PV patient IgG do contain antibodies that sterically hinder Dsg3 adhesion. Nevertheless, the actual concentration or affinity of those specific antibodies may vary among patients. As a result, it is possible that some PV IgG may cause a substantial steric hindrance effect rather than Dsg3 depletion and desmosomal disassembly, or vice versa [53]. Previously reported observations of nearly intact (not-disassembled) half-split desmosomes in PV patients and model mice supports this idea [49], [50].

**Figure 8:**
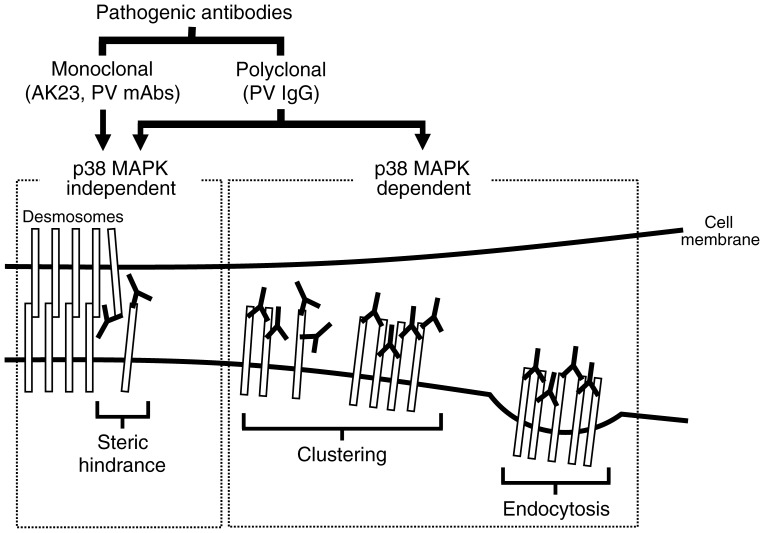
Model for PV pathogenicity. Monoclonal pathogenic antibodies, such as AK23 and monovalent PV mAbs (scFv) cloned from patients, cause loss of adhesion primarily through steric hindrance, which does not require signaling through p38 MAPK. In contrast, PV IgG induce two separate pathogenic events. In addition to the steric hindrance caused by a portion of antibodies contained in PV IgG, the polyclonal nature of PV IgG causes clustering and endocytosis of Dsg3 in a p38 MAPK dependent manner.

In summary, the results presented here demonstrate that the polyclonal nature of PV IgG contributes significantly to PV pathogenicity by causing clustering and endocytosis of Dsg3 through a p38 MAPK dependent mechanism (Fig. 8). Importantly, these findings clarify for the first time that Dsg3 depletion involving p38 MAPK and steric hindrance are two separate events caused by PV IgG. Moreover, clustering and endocytosis of Dsg3 appears to be sufficient to cause loss of cell-cell adhesion, as shown in the cases of PV IgG lacking antibodies against the adhesive region of Dsg3. In contrast, monoclonal pathogenic antibodies cause loss of adhesion primarily through steric hindrance. Furthermore, loss of desmosomal adhesion itself due to steric hindrance does not require signaling through p38 MAPK, but may activate this pathway subsequent to detachment [29]. These findings underscore the importance of using both patient derived IgG and monoclonal Dsg3 reagents to evaluate pemphigus pathomechanisms and possible therapeutic interventions.

## Methods

### Ethics Statement

Approvals for use of human IgG and skin samples were obtained from appropriate review boards at Emory University and the University of Pennsylvania. Specifically, studies used existing, otherwise discarded, de-identified human skin samples that were obtained for clinical purposes during skin surgery repair procedures. These studies were approved for human subjects research exemption (informed consent not required) by the Institutional Review Board at the University of Pennsylvania under the United States Health and Human Services Code of Federal Regulations 46.101(b)(4).

### Cells and Culture Conditions

Normal human epidermal keratinocytes were isolated from neonatal foreskin as previously described [30]. Cells were propagated in low calcium medium (Medium 154, Invitrogen, Carlsbad, CA, or KGM-Gold, Lonza, Walkersville, MD) with growth supplements (HKGS, Invitrogen, or KGM-Gold SingleQuot Kit, Lonza). Keratinocytes, no later than passage 3, were then switched into medium containing 0.6 mM calcium chloride for 16–18 hours before experimental manipulations.

### Antibodies and Reagents

Normal human (NH) IgG was purchased from Invitrogen. PV sera were kind gifts from Dr. Masayuki Amagai (Keio University, Tokyo), Dr. John Stanley (University of Pennsylvania, Philadelphia, PA), and Dr. Robert Swerlick (Emory University, Atlanta, GA). PV sera used in this study recognized Dsg3, but not Dsg1, as determined by ELISA. The majority of the experiments shown in this manuscript were conducted using IgG preparations from a single patient (#4030 ELISA score: Dsg3 179.3, Dsg1 2.6) However, similar results (ie. Dsg3 clustering) were obtained using at least 5 additional patient sera (not shown, see also [30], [31]). IgG was purified from PV sera using Melon Gel IgG Purification Resins and Kits (Thermo Fisher Scientific, Rockford, IL) according to the manufacturer’s protocol. Monoclonal mouse anti-Dsg3 antibodies AK15, AK19, and AK23, and pathogenic and nonpathogenic monovalent PV mAbs (scFv), were generated as previously described [17], [18]. AK19 and AK23 are pathogenic antibodies, whose epitopes were previously shown to locate in residues 87-161 and 1–63 of the extracellular domain of mDsg3, respectively [18]. The epitope of AK15, which lacks pathogenic activity, was mapped to residues 195–402. Other antibodies used were as follows: a rabbit anti-desmoplakin antibody (NW6 from Dr. Kathleen Green, Northwestern University), a rabbit anti-gamma catenin (plakoglobin) antibody (H-80, Santa Cruz Biotechnology, Santa Cruz, CA) anti-IL-2 receptor (IL-2R) IgG2a (IL-2R_IgG2a_) from 7G7B6 mouse hybridoma (American Type Culture Collection, Manasssas, VA), mouse anti-IL-2R IgG1 (R&D Systems, Minneapolis, MN), a polyclonal goat antibody against mouse IgG (Thermo Fisher Scientific), and a rat monoclonal anti-HA antibody (3F10, Roche, Nutley, NJ). Secondary antibodies conjugated to Alexa Fluors (Invitrogen) were used for double- and triple-label experiments. The tyrosine kinase inhibitor genistein was purchased from Sigma-Aldrich (St.Louis, MO) and used at 50 µM. The p38 MAPK inhibitor SB202190 was purchased from Calbiochem (La Jolla, CA) and used at 20 µM. Treatment of cells with either genistein or SB202190 was conducted 1 hour prior to antibody addition and remained in the medium for the duration of the experiment.

### Adenovirus Expression

A chimeric protein comprising the IL-2R extracellular domain and the Dsg3 cytoplasmic tail (IL-2R-Dsg3_cyto_) was generated as described previously [26]. In addition, two chimeric constructs were generated. Specifically, sequences encoding a FLAG epitope tagged Dsg3 cytoplasmic tail lacking the last 133 amino acids that include the repeating unit domain (RUD) were fused to the extracellular domain of the IL-2R [54] (IL-2R-Dsg3_cytoΔ866_). A similar chimera was generated to comprise the Dsg3 cytoplasmic tail lacking the last 284 amino acids, including both the RUD and intracellular cadherin-specific domain (ICS) (IL-2R-Dsg3_cytoΔ715_). The PCR primers used to generate these constructs were as follows: IL-2R-Dsg3_cytoΔ866_, 5-primer, 5′- GCCATGACTAGTATGTGACTGTGGGGCAGGTTCTACT; 3-primer, 5′- CCGGATATCCTACTTATCGTCGTCATCCTTGTAATCTTCACCATCAACACCAAGGCTTAT; IL-2R-Dsg3_cytoΔ715_, 5-primer, 5′- GCCATGACTAGTATGTGACTGTGGGGCAGGTTCTACT; 3-primer, 5′-CCGGATATCCTACTTATCGTCGTCATCCTTGTAATCGCCTTCCACCGCTGTGCCTCTGGC. All constructs were verified by DNA sequencing analysis, western blotting, and immunofluorescence analysis. Adenoviruses carrying each chimeric construct were produced using the pAdEeasy adenovirus-packaging system as described previously [55], [56].

### Immunofluorescence

Keratinocytes were seeded onto glass coverslips and allowed to grow to desired confluence. Cells were then switched into medium containing 0.6 mM calcium for 16–18 hours before treatment with NH IgG, PV IgG, AK23, or PV mAbs for varying amounts of time. For the 0 hour time-point, cells were incubated with antibodies at 4°C for 30 minutes. NH IgG or PV IgG was added to cell culture medium at 100–150 µg/ml, while AK23 was added at 10–15 µg/ml, and PV mAbs at 100 µg/ml. On completion of the incubation at 37°C, cells were washed three times with phosphate-buffered saline (PBS), and then fixed on ice using −20°C methanol for 2 minutes and stained with antibodies indicated above. Widefield microscopy was performed using a Leica DMR-E microscope (Leica, Wetzler, Germany) equipped with narrow bandpass filters and a Hamamatsu Orca camera (Hamamatsu, Bridgewater, NJ). Images were captured and processed using Simple PCI software (Hamamatsu Corporation, Sewickley, PA). Super-resolution microscopy was performed using a Nikon N-SIM system on an Eclipse Ti-E microscope equipped with a 100×/1.49NA oil immersion objective, 561 nm and 488 nm solid-state lasers, and a DU-897 EM-CCD camera (Andor). Images were acquired in 3D structured-illumination microscopy mode and reconstructed using NISElements software with the N-SIM module (version 3.22; Nikon).

### Pulse-chase Assay

Biotinylation of AK23 and IL-2R_IgG2a_ was carried out using EZ-Link Sulfo-NHS-SS-Biotin (Thermo Fisher Scientific) following the manufacturer’s instructions. Keratinocytes were seeded and grown on glass coverslips until reaching desired confluence. Cells were switched into 0.6 mM calcium containing medium for 16–18 hours before treatment with biotinylated AK23 (AK23-biotin) for 30 minutes on ice to label cell surface Dsg3. Cells were then rinsed three times with PBS to remove unbound AK23-biotin, and incubated with antibodies for 6 hours at 37°C. After fixing on ice with 4% paraformaldehyde (Electron Microscopy Sciences, Hatfield, PA) for 10 minutes without permeabilization, cells were stained with Alexa Fluor 555-conjugated strepavidin (Invitrogen) to detect remaining cell surface levels of Dsg3. In the pulse-chase study using Dsg3 chimeras, cells were infected with adenovirus expressing each chimera prior to the calcium switch, and chimeras expressed on the cell surface were labeled with biotinylated IL-2R_IgG2a_.

### Fluorescence Intensity Measurements

To measure Dsg3 clustering by polyclonal antibodies, analysis of Dsg3 fluorescent intensity along cell-cell borders was analyzed. Briefly, using ImageJ software (NIH, Bethesda, MD), lines were drawn along cell borders and the level of fluorescence intensity at each pixel along the lines was obtained (Fig. 2I). To calculate the number of pixels between peaks, the number of pixels in the line segment was divided by the number of peaks in the line segment. Twenty line segments were taken for each condition to obtain average number of pixels between peaks. Cell surface fluorescence intensity (total grey) was measured using Simple PCI software. To quantitate loss of cell surface chimera, the percentage of chimera remaining on the cell surface was calculated against total expression based on measurements of fluorescence intensity.

### Electron Microscopy

Keratinocytes were prepared on coverslips as above and treated with either NH IgG, PV IgG, or AK23 for 24 hours at 37°C. Cells were fixed with 2.5% glutaraldehyde in 0.1 M cacodylate. Samples were then processed for conventional electron microscopy (Robert P. Apkarian Integrated Electron Microscopy Core).

### Cell Surface Biotinylation and Western Blotting Analysis

Keratinocytes were grown to 70% confluency in 12-well tissue culture plates. Cells were switched to medium with 0.6 mM calcium 16–18 hours prior to biotinylation. Cells were rinsed with PBS three times and labeled with EZ-Link Sulfo-NHS-SS-Biotin at 1 mg/ml in PBS for 1 hour on ice. Excess biotin was quenched by washing three times with PBS containing 50 mM ammonium chloride for 5 minutes each on ice, followed by rinsing with PBS four times. Cells were then treated with either NH IgG, PV IgG, IL-2R, or AK23 in medium containing 0.6 mM calcium for 24 hours at 37°C. Cells were lysed using cytoskeleton (CSK) buffer (50 mM NaCl, 10 mM PIPES, 3 mM MgCl_2_, 300 mM Sucrose, 10 mM NaF, 10 mM NaP_2_O_7_, pH 6.8) containing 1% Triton X-100 (Roche) and protease inhibitor cocktail tablets (Roche). Lysate of cells immediately after biotinylation was used as a control at time 0. After centrifugation at 14,000×g, the supernatant was recovered and incubated with streptavidin agarose resin (Thermo Fisher Scientific) for 1 hour at 4°C. Cell surface biotinylated proteins were captured by centrifugation, and the resin was rinsed four times with CSK buffer. Biotinylated proteins were released in Laemmli buffer containing β-mercaptoethanol at 95°C and then resolved by 7.5% SDS-PAGE, and transferred to a nitrocellulose membrane according to standard protocols. The membranes were analyzed for Dsg3 using an anti-Dsg3 antibody mixture containing AK15 and a mouse anti-Dsg3 antibody (5G11, Invitrogen). A mouse anti-E-cadherin antibody (BD Biosciences, San Jose, CA) was used as a loading control.

### Dispase-based Dissociation Assay

Keratinocytes were grown to 100% confluence in 12-well tissue culture plates. Cells were switched to media containing 0.6 mM calcium 16–18 hours before the addition of NH or PV IgG at 100–150 µg/ml, or AK23 at 10–15 µg/ml. Recombinant exfoliative toxin A (ETA, 0.25 µg/ml) produced in E.coli, which specifically cleaves Dsg1, was added 2 hours before the assay. After 6-hour incubation with the antibody, cells were rinsed with PBS and incubated with 1 U/ml dispase (Roche) for 1 hour. Cell sheets released from the plate were rinsed with PBS, and then subjected to mechanical stress by pipetting with a 1 ml pipette tip. Fragments of the sheets were fixed with formaldehyde and stained with methylene blue (Sigma), and counted using a dissecting microscope.

### Human Skin Explant Injections

Normal human explants were washed thoroughly with PBS and cut into 5 mm^2^ sections after removing subcutaneous adipose tissue. Organ-cultured tissue sections were pretreated by intradermal injection with DMSO (50 µl), 6.25 µg genistein (Sigma-Aldrich) or 5.0 µg SB202190 (Calbiochem) in 50 µl DMSO for 2 hours. AK23 (25–50 µg) or PV IgG (160–400 µg) was injected intradermally in the presence of 0.8 µg ETA. Skin sections were then transferred to Transwell inserts (Corning) with defined keratinocyte SFM medium (Invitrogen) containing 1.2 mM CaCl_2_. The skin sections were processed for widefield or structured illumination immunofluorescence and histology (HE staining) 16 hours post antibody injection. PV IgG and AK23 binding were detected with goat anti-human Alexa Fluor 594 and goat anti-mouse Alexa Fluor 555 secondary antibodies, respectively.

### Statistics

Statistical analysis of fluorescence intensity measurements was performed using Kruskal-Wallis one-way analysis of variance on ranks with multiple comparisons performed by Dunn’s method with a significance level of 0.05. Statistical analysis of dispase-based dissociation assay was performed using a *t*-test assuming unequal variances with a significance level of 0.05.

## Supporting Information

Figure S1 **Monoclonal antibodies used at a high dose do not cause Dsg3 clustering.** Desmoplakin (DP) localization was unchanged after addition of NH IgG. After addition of PV IgG and a 6 hr incubation at 37°C, both IgG and DP staining became discontinuous. In contrast, IgG and DP staining remained in a linear and punctate pattern after a high dose (100 µg/ml) treatment with monoclonal antibodies AK15 (non-pathogenic) and AK23 (pathogenic). Scale bar, 10 µm.
(TIF)

Figure S2 **Pathogenic human Dsg3 antibodies cloned from PV patients do not cause clustering of surface Dsg3.** Pathogenic and non-pathogenic monovalent mAbs (scFv) bound to keratinocyte cell-cell borders and were detectable for up to 24 h (A, B, G, H). Immunofluorescence detection of Dsg3 (C, D, I, J) and desmoplakin (E, F, K, L) revealed little or no change in desmosomal protein organization irrespective of the pathogenic nature of the antibodies. Scale bar, 10 µm.
(TIF)

Figure S3 **AK23-biotin pulse-label does not induce changes in Dsg3 distribution.** Fluorescence intensity along borders of cells fixed prior to AK23 addition were compared to cells pulse-labeled with AK23 at 4°C for 30 min followed by a 6 hr chase period at 37°C. The results indicate that the AK23-biotin labeling procedure used in this study did not induce changes in Dsg3 distribution as no detectable change in pixel number between peaks could be detected over the 6 hr time course.
(TIF)

Figure S4 **PV IgG directed against the Dsg3 EC1 domain are not required to cause blistering** ***in vivo*** **.** Clinical presentation of lower back epidermal blisters (A) and histopathology (B) of patient PV IgG(b). Note that this patient lacks IgG directed against the Dsg3 EC1 domain (see Figure 7).
(TIF)

Figure S5 **p38MAPK inhibition prevents Dsg3 clustering induced by PV IgG lacking EC1 antibodies.** Cell surface Dsg3 was monitored using biotinylated AK23 followed by the addition of NH IgG, PV IgG and PV IgG (a). Cells were treated with the p38MAPK inhibitor SB202190 prior and during the addition of IgG. SB202190 prevented Dsg3 clustering induced by both PV IgG and PV IgG (a), the latter which only contains antibodies directed against domains EC3–4.
(TIF)

## References

[1] Delva E, Tucker DK, Kowalczyk AP (2009) The desmosome. Cold Spring Harb Perspect Biol 1: a002543. 20066089 10.1101/cshperspect.a002543 PMC2742091

[2] Getsios S, Huen AC, Green KJ (2004) Working out the strength and flexibility of desmosomes. Nat Rev Mol Cell Biol 5: 271–281. 15071552 10.1038/nrm1356

[3] Yin T, Green KJ (2004) Regulation of desmosome assembly and adhesion. Semin Cell Dev Biol 15: 665–677. 15561586 10.1016/j.semcdb.2004.09.005

[4] Amagai M, Stanley JR (2012) Desmoglein as a target in skin disease and beyond. J Invest Dermatol 132: 776–784. 22189787 10.1038/jid.2011.390 PMC3279627

[5] Brooke MA, Nitoiu D, Kelsell DP (2012) Cell-cell connectivity: desmosomes and disease. J Pathol 226: 158–171. 21989576 10.1002/path.3027

[6] Green KJ, Getsios S, Troyanovsky S, Godsel LM (2010) Intercellular junction assembly, dynamics, and homeostasis. Cold Spring Harb Perspect Biol 2: a000125. 20182611 10.1101/cshperspect.a000125 PMC2828282

[7] Amagai M (2010) Autoimmune and infectious skin diseases that target desmogleins. Proc Jpn Acad Ser B Phys Biol Sci 86: 524–537. 10.2183/pjab.86.524 PMC3108298 20467217

[8] Amagai M, Klaus-Kovtun V, Stanley JR (1991) Autoantibodies against a novel epithelial cadherin in pemphigus vulgaris, a disease of cell adhesion. Cell 67: 869–877. 1720352 10.1016/0092-8674(91)90360-b

[9] Anhalt GJ, Diaz LA (2004) Pemphigus vulgaris–a model for cutaneous autoimmunity. J Am Acad Dermatol 51: S20–21. 15243495 10.1016/j.jaad.2004.01.011

[10] Payne AS, Hanakawa Y, Amagai M, Stanley JR (2004) Desmosomes and disease: pemphigus and bullous impetigo. Curr Opin Cell Biol 16: 536–543. 15363804 10.1016/j.ceb.2004.07.006

[11] Waschke J (2008) The desmosome and pemphigus. Histochem Cell Biol 130: 21–54. 18386043 10.1007/s00418-008-0420-0 PMC2413110

[12] Getsios S, Waschke J, Borradori L, Hertl M, Muller EJ (2010) From cell signaling to novel therapeutic concepts: international pemphigus meeting on advances in pemphigus research and therapy. J Invest Dermatol 130: 1764–1768. 20548314 10.1038/jid.2010.111

[13] Sharma P, Mao X, Payne AS (2007) Beyond steric hindrance: the role of adhesion signaling pathways in the pathogenesis of pemphigus. J Dermatol Sci 48: 1–14. 17574391 10.1016/j.jdermsci.2007.05.005

[14] Heupel WM, Zillikens D, Drenckhahn D, Waschke J (2008) Pemphigus vulgaris IgG directly inhibit desmoglein 3-mediated transinteraction. J Immunol 181: 1825–1834. 18641320 10.4049/jimmunol.181.3.1825

[15] Shapiro L, Weis WI (2009) Structure and biochemistry of cadherins and catenins. Cold Spring Harb Perspect Biol 1: a003053. 20066110 10.1101/cshperspect.a003053 PMC2773639

[16] Mascaro JM Jr, Espana A, Liu Z, Ding X, Swartz SJ, et al (1997) Mechanisms of acantholysis in pemphigus vulgaris: role of IgG valence. Clin Immunol Immunopathol 85: 90–96. 9325074 10.1006/clin.1997.4408

[17] Payne AS, Ishii K, Kacir S, Lin C, Li H, et al (2005) Genetic and functional characterization of human pemphigus vulgaris monoclonal autoantibodies isolated by phage display. J Clin Invest 115: 888–899. 15841178 10.1172/JCI24185 PMC1070425

[18] Tsunoda K, Ota T, Aoki M, Yamada T, Nagai T, et al (2003) Induction of pemphigus phenotype by a mouse monoclonal antibody against the amino-terminal adhesive interface of desmoglein 3. J Immunol 170: 2170–2178. 12574390 10.4049/jimmunol.170.4.2170

[19] Berkowitz P, Hu P, Liu Z, Diaz LA, Enghild JJ, et al (2005) Desmosome signaling. Inhibition of p38MAPK prevents pemphigus vulgaris IgG-induced cytoskeleton reorganization. J Biol Chem 280: 23778–23784. 15840580 10.1074/jbc.M501365200

[20] Berkowitz P, Hu P, Warren S, Liu Z, Diaz LA, et al (2006) p38MAPK inhibition prevents disease in pemphigus vulgaris mice. Proc Natl Acad Sci U S A 103: 12855–12860. 16908851 10.1073/pnas.0602973103 PMC1568937

[21] Chernyavsky AI, Arredondo J, Kitajima Y, Sato-Nagai M, Grando SA (2007) Desmoglein versus non-desmoglein signaling in pemphigus acantholysis: characterization of novel signaling pathways downstream of pemphigus vulgaris antigens. J Biol Chem 282: 13804–13812. 17344213 10.1074/jbc.M611365200

[22] Seishima M, Iwasaki-Bessho Y, Itoh Y, Nozawa Y, Amagai M, et al (1999) Phosphatidylcholine-specific phospholipase C, but not phospholipase D, is involved in pemphigus IgG-induced signal transduction. Arch Dermatol Res 291: 606–613. 10638334 10.1007/s004030050462

[23] Waschke J, Spindler V, Bruggeman P, Zillikens D, Schmidt G, et al (2006) Inhibition of Rho A activity causes pemphigus skin blistering. J Cell Biol 175: 721–727. 17130286 10.1083/jcb.200605125 PMC2064672

[24] Williamson L, Raess NA, Caldelari R, Zakher A, de Bruin A, et al (2006) Pemphigus vulgaris identifies plakoglobin as key suppressor of c-Myc in the skin. EMBO J 25: 3298–3309. 16871158 10.1038/sj.emboj.7601224 PMC1523185

[25] Sanchez-Carpintero I, Espana A, Pelacho B, Lopez Moratalla N, Rubenstein DS, et al (2004) In vivo blockade of pemphigus vulgaris acantholysis by inhibition of intracellular signal transduction cascades. Br J Dermatol 151: 565–570. 15377341 10.1111/j.1365-2133.2004.06147.x

[26] Delva E, Jennings JM, Calkins CC, Kottke MD, Faundez V, et al (2008) Pemphigus vulgaris IgG-induced desmoglein-3 endocytosis and desmosomal disassembly are mediated by a clathrin- and dynamin-independent mechanism. J Biol Chem 283: 18303–18313. 18434319 10.1074/jbc.M710046200 PMC2440613

[27] Spindler V, Waschke J (2011) Role of Rho GTPases in desmosomal adhesion and pemphigus pathogenesis. Ann Anat 193: 177–180. 21441018 10.1016/j.aanat.2011.02.003

[28] Jolly PS, Berkowitz P, Bektas M, Lee HE, Chua M, et al (2010) p38MAPK signaling and desmoglein-3 internalization are linked events in pemphigus acantholysis. J Biol Chem 285: 8936–8941. 20093368 10.1074/jbc.M109.087999 PMC2838315

[29] Mao X, Sano Y, Park JM, Payne AS (2011) p38 MAPK activation is downstream of the loss of intercellular adhesion in pemphigus vulgaris. J Biol Chem 286: 1283–1291. 21078676 10.1074/jbc.M110.172874 PMC3020736

[30] Calkins CC, Setzer SV, Jennings JM, Summers S, Tsunoda K, et al (2006) Desmoglein endocytosis and desmosome disassembly are coordinated responses to pemphigus autoantibodies. J Biol Chem 281: 7623–7634. 16377623 10.1074/jbc.M512447200

[31] Jennings JM, Tucker DK, Kottke MD, Saito M, Delva E, et al (2011) Desmosome disassembly in response to pemphigus vulgaris IgG occurs in distinct phases and can be reversed by expression of exogenous Dsg3. J Invest Dermatol 131: 706–718. 21160493 10.1038/jid.2010.389 PMC3235416

[32] Mao X, Choi EJ, Payne AS (2009) Disruption of desmosome assembly by monovalent human pemphigus vulgaris monoclonal antibodies. J Invest Dermatol 129: 908–918. 19037235 10.1038/jid.2008.339 PMC2743719

[33] Yamamoto Y, Aoyama Y, Shu E, Tsunoda K, Amagai M, et al (2007) Anti-desmoglein 3 (Dsg3) monoclonal antibodies deplete desmosomes of Dsg3 and differ in their Dsg3-depleting activities related to pathogenicity. J Biol Chem 282: 17866–17876. 17428808 10.1074/jbc.M607963200

[34] Xiao K, Garner J, Buckley KM, Vincent PA, Chiasson CM, et al (2005) p120-Catenin regulates clathrin-dependent endocytosis of VE-cadherin. Mol Biol Cell 16: 5141–5151. 16120645 10.1091/mbc.E05-05-0440 PMC1266414

[35] Chiasson CM, Wittich KB, Vincent PA, Faundez V, Kowalczyk AP (2009) p120-catenin inhibits VE-cadherin internalization through a Rho-independent mechanism. Mol Biol Cell 20: 1970–1980. 19211843 10.1091/mbc.E08-07-0735 PMC2663933

[36] Sekiguchi M, Futei Y, Fujii Y, Iwasaki T, Nishikawa T, et al (2001) Dominant autoimmune epitopes recognized by pemphigus antibodies map to the N-terminal adhesive region of desmogleins. J Immunol 167: 5439–5448. 11673563 10.4049/jimmunol.167.9.5439

[37] Yokouchi M, Saleh MA, Kuroda K, Hachiya T, Stanley JR, et al (2009) Pathogenic epitopes of autoantibodies in pemphigus reside in the amino-terminal adhesive region of desmogleins which are unmasked by proteolytic processing of prosequence. J Invest Dermatol 129: 2156–2166. 19340014 10.1038/jid.2009.61 PMC2813511

[38] Mahoney MG, Wang Z, Rothenberger K, Koch PJ, Amagai M, et al (1999) Explanations for the clinical and microscopic localization of lesions in pemphigus foliaceus and vulgaris. J Clin Invest 103: 461–468. 10021453 10.1172/JCI5252 PMC408100

[39] Stanley JR, Amagai M (2006) Pemphigus, bullous impetigo, and the staphylococcal scalded-skin syndrome. N Engl J Med 355: 1800–1810. 17065642 10.1056/NEJMra061111

[40] Caldelari R, de Bruin A, Baumann D, Suter MM, Bierkamp C, et al (2001) A central role for the armadillo protein plakoglobin in the autoimmune disease pemphigus vulgaris. J Cell Biol 153: 823–834. 11352942 10.1083/jcb.153.4.823 PMC2192383

[41] Muller EJ, Williamson L, Kolly C, Suter MM (2008) Outside-in signaling through integrins and cadherins: a central mechanism to control epidermal growth and differentiation? J Invest Dermatol 128: 501–516. 18268536 10.1038/sj.jid.5701248

[42] Aoyama Y, Kitajima Y (1999) Pemphigus vulgaris-IgG causes a rapid depletion of desmoglein 3 (Dsg3) from the Triton X-100 soluble pools, leading to the formation of Dsg3-depleted desmosomes in a human squamous carcinoma cell line, DJM-1 cells. J Invest Dermatol 112: 67–71. 9886266 10.1046/j.1523-1747.1999.00463.x

[43] Aoyama Y, Nagai M, Kitajima Y (2010) Binding of pemphigus vulgaris IgG to antigens in desmosome core domains excludes immune complexes rather than directly splitting desmosomes. Br J Dermatol 162: 1049–1055. 20222919 10.1111/j.1365-2133.2010.09672.x

[44] Shu E, Yamamoto Y, Aoyama Y, Kitajima Y (2007) Intraperitoneal injection of pemphigus vulgaris-IgG into mouse depletes epidermal keratinocytes of desmoglein 3 associated with generation of acantholysis. Arch Dermatol Res 299: 165–167. 17431647 10.1007/s00403-007-0754-9

[45] Sato M, Aoyama Y, Kitajima Y (2000) Assembly pathway of desmoglein 3 to desmosomes and its perturbation by pemphigus vulgaris-IgG in cultured keratinocytes, as revealed by time-lapsed labeling immunoelectron microscopy. Lab Invest 80: 1583–1592. 11045575 10.1038/labinvest.3780168

[46] Kawasaki H, Tsunoda K, Hata T, Ishii K, Yamada T, et al (2006) Synergistic pathogenic effects of combined mouse monoclonal anti-desmoglein 3 IgG antibodies on pemphigus vulgaris blister formation. J Invest Dermatol 126: 2621–2630. 16841036 10.1038/sj.jid.5700450

[47] Schulze K, Galichet A, Sayar BS, Scothern A, Howald D, et al (2012) An adult passive transfer mouse model to study desmoglein 3 signaling in pemphigus vulgaris. J Invest Dermatol 132: 346–355. 21956125 10.1038/jid.2011.299 PMC3258361

[48] Ishii K, Harada R, Matsuo I, Shirakata Y, Hashimoto K, et al (2005) In vitro keratinocyte dissociation assay for evaluation of the pathogenicity of anti-desmoglein 3 IgG autoantibodies in pemphigus vulgaris. J Invest Dermatol 124: 939–946. 15854034 10.1111/j.0022-202X.2005.23714.x

[49] Shimizu A, Ishiko A, Ota T, Tsunoda K, Amagai M, et al (2004) IgG binds to desmoglein 3 in desmosomes and causes a desmosomal split without keratin retraction in a pemphigus mouse model. J Invest Dermatol 122: 1145–1153. 15140217 10.1111/j.0022-202X.2004.22426.x

[50] Wang W, Amagai M, Ishiko A (2009) Desmosome splitting is a primary ultrastructural change in the acantholysis of pemphigus. J Dermatol Sci 54: 59–61. 19097754 10.1016/j.jdermsci.2008.10.010

[51] Shimizu A, Ishiko A, Ota T, Saito H, Oka H, et al (2005) In vivo ultrastructural localization of the desmoglein 3 adhesive interface to the desmosome mid-line. J Invest Dermatol 124: 984–989. 15854040 10.1111/j.0022-202X.2005.23706.x

[52] Oktarina DA, van der Wier G, Diercks GF, Jonkman MF, Pas HH (2011) IgG-induced clustering of desmogleins 1 and 3 in skin of patients with pemphigus fits with the desmoglein nonassembly depletion hypothesis. Br J Dermatol 165: 552–562. 21692763 10.1111/j.1365-2133.2011.10463.x

[53] Waschke J, Bruggeman P, Baumgartner W, Zillikens D, Drenckhahn D (2005) Pemphigus foliaceus IgG causes dissociation of desmoglein 1-containing junctions without blocking desmoglein 1 transinteraction. J Clin Invest 115: 3157–3165. 16211092 10.1172/JCI23475 PMC1242188

[54] LaFlamme SE, Thomas LA, Yamada SS, Yamada KM (1994) Single subunit chimeric integrins as mimics and inhibitors of endogenous integrin functions in receptor localization, cell spreading and migration, and matrix assembly. J Cell Biol 126: 1287–1298. 8063864 10.1083/jcb.126.5.1287 PMC2120158

[55] Setzer SV, Calkins CC, Garner J, Summers S, Green KJ, et al (2004) Comparative analysis of armadillo family proteins in the regulation of a431 epithelial cell junction assembly, adhesion and migration. J Invest Dermatol 123: 426–433. 15304078 10.1111/j.0022-202X.2004.23319.x

[56] Xiao K, Allison DF, Kottke MD, Summers S, Sorescu GP, et al (2003) Mechanisms of VE-cadherin processing and degradation in microvascular endothelial cells. J Biol Chem 278: 19199–19208. 12626512 10.1074/jbc.M211746200

