# Supplementary figures and images for: Signaling Dependent and Independent Mechanisms in Pemphigus Vulgaris Blister Formation
Source: PLoS One. 2012 Dec 3;7(12):e50696. doi: 10.1371/journal.pone.0050696 (PMC3513318; doi:10.1371/journal.pone.0050696)

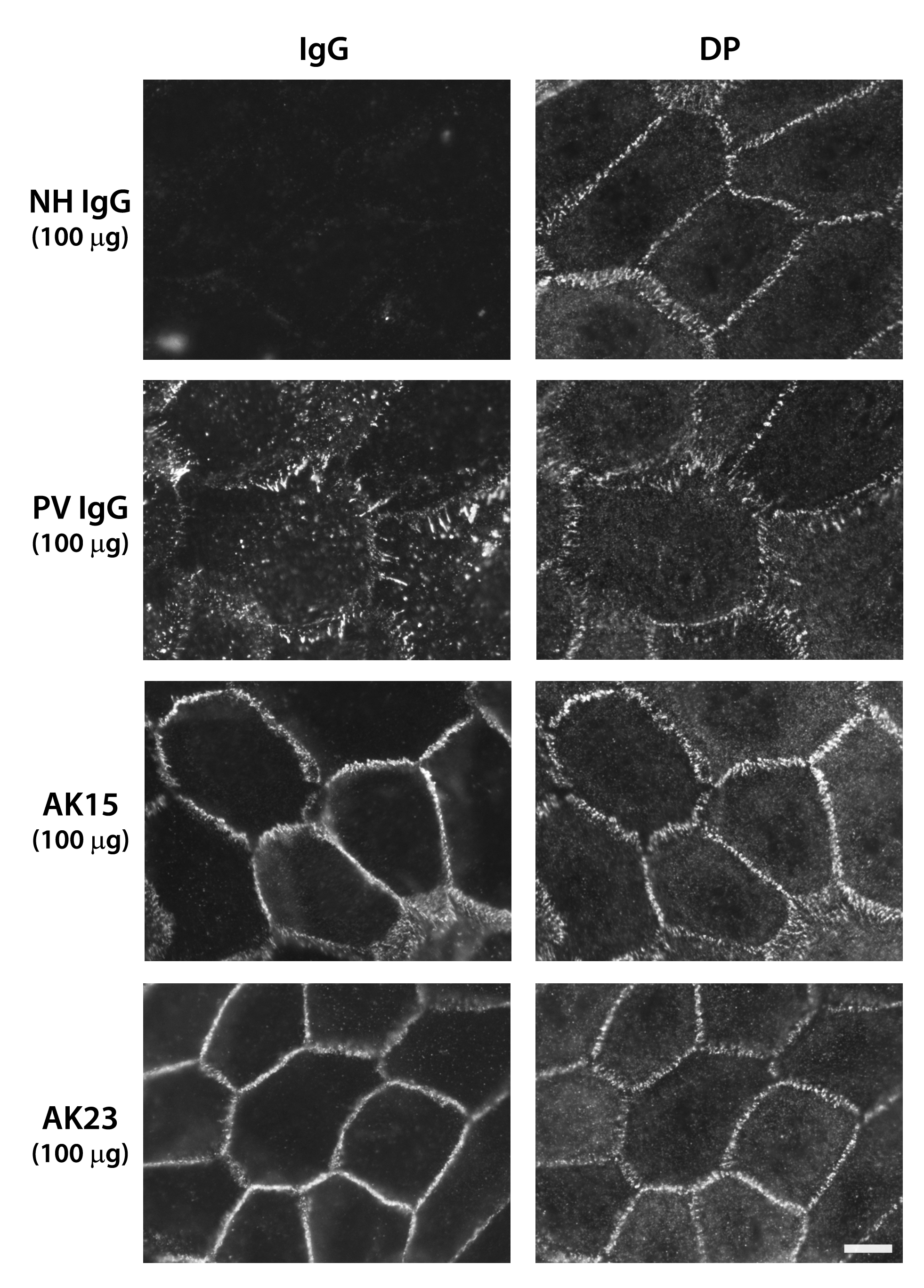

Supplement: Figure S1 — Monoclonal antibodies used at a high dose do not cause Dsg3 clustering. Desmoplakin (DP) localization was unchanged after addition of NH IgG. After addition of PV IgG and a 6 hr incubation at 37°C, both IgG and DP staining became discontinuous. In contrast, IgG and DP staining remained in a linear and punctate pattern after a high dose (100 µg/ml) treatment with monoclonal antibodies AK15 (non-pathogenic) and AK23 (pathogenic). Scale bar, 10 µm. (TIF) [file pone.0050696.s001.tif]

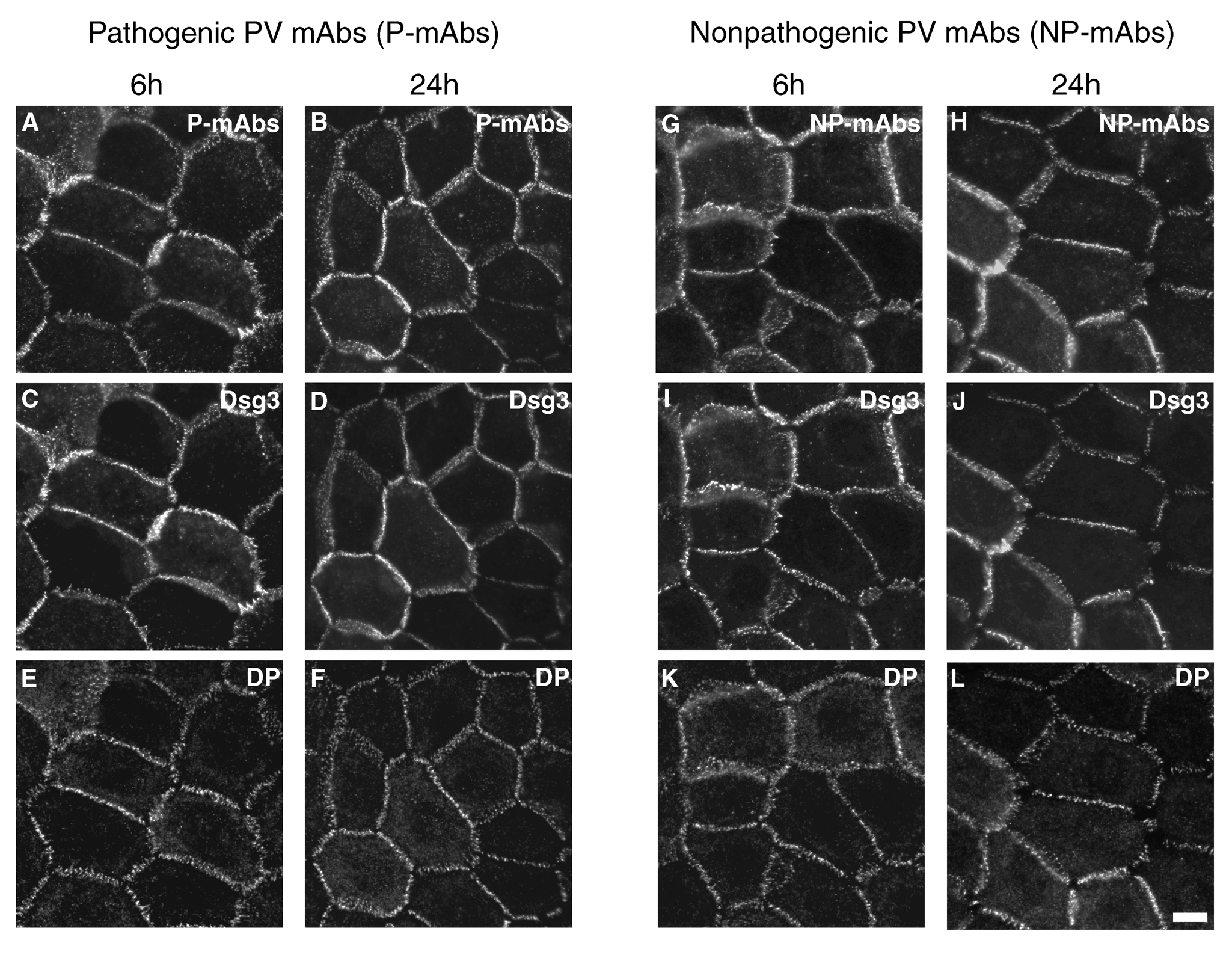

Supplement: Figure S2 — Pathogenic human Dsg3 antibodies cloned from PV patients do not cause clustering of surface Dsg3. Pathogenic and non-pathogenic monovalent mAbs (scFv) bound to keratinocyte cell-cell borders and were detectable for up to 24 h (A, B, G, H). Immunofluorescence detection of Dsg3 (C, D, I, J) and desmoplakin (E, F, K, L) revealed little or no change in desmosomal protein organization irrespective of the pathogenic nature of the antibodies. Scale bar, 10 µm. (TIF) [file pone.0050696.s002.tif]

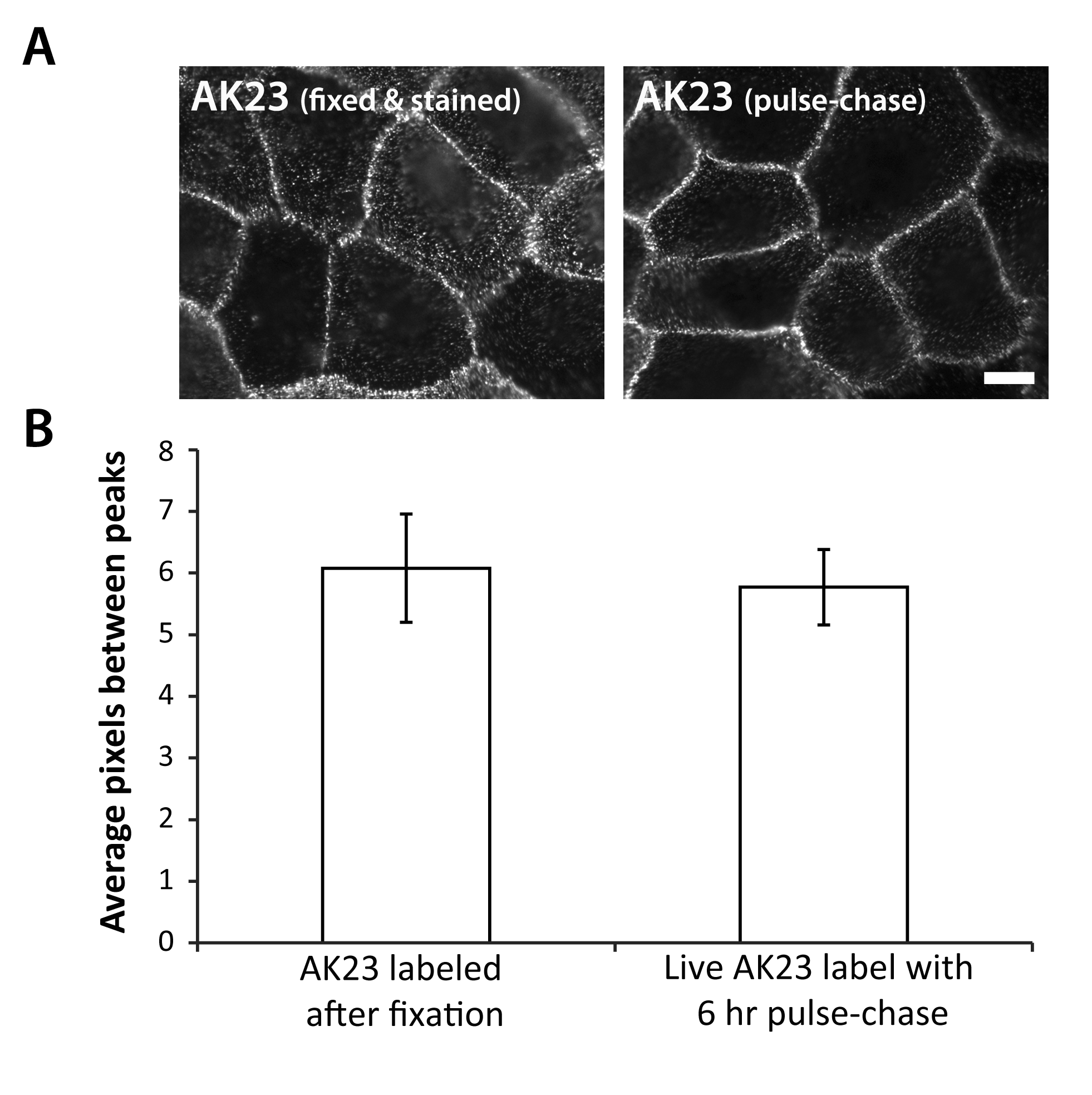

Supplement: Figure S3 — AK23-biotin pulse-label does not induce changes in Dsg3 distribution. Fluorescence intensity along borders of cells fixed prior to AK23 addition were compared to cells pulse-labeled with AK23 at 4°C for 30 min followed by a 6 hr chase period at 37°C. The results indicate that the AK23-biotin labeling procedure used in this study did not induce changes in Dsg3 distribution as no detectable change in pixel number between peaks could be detected over the 6 hr time course. (TIF) [file pone.0050696.s003.tif]

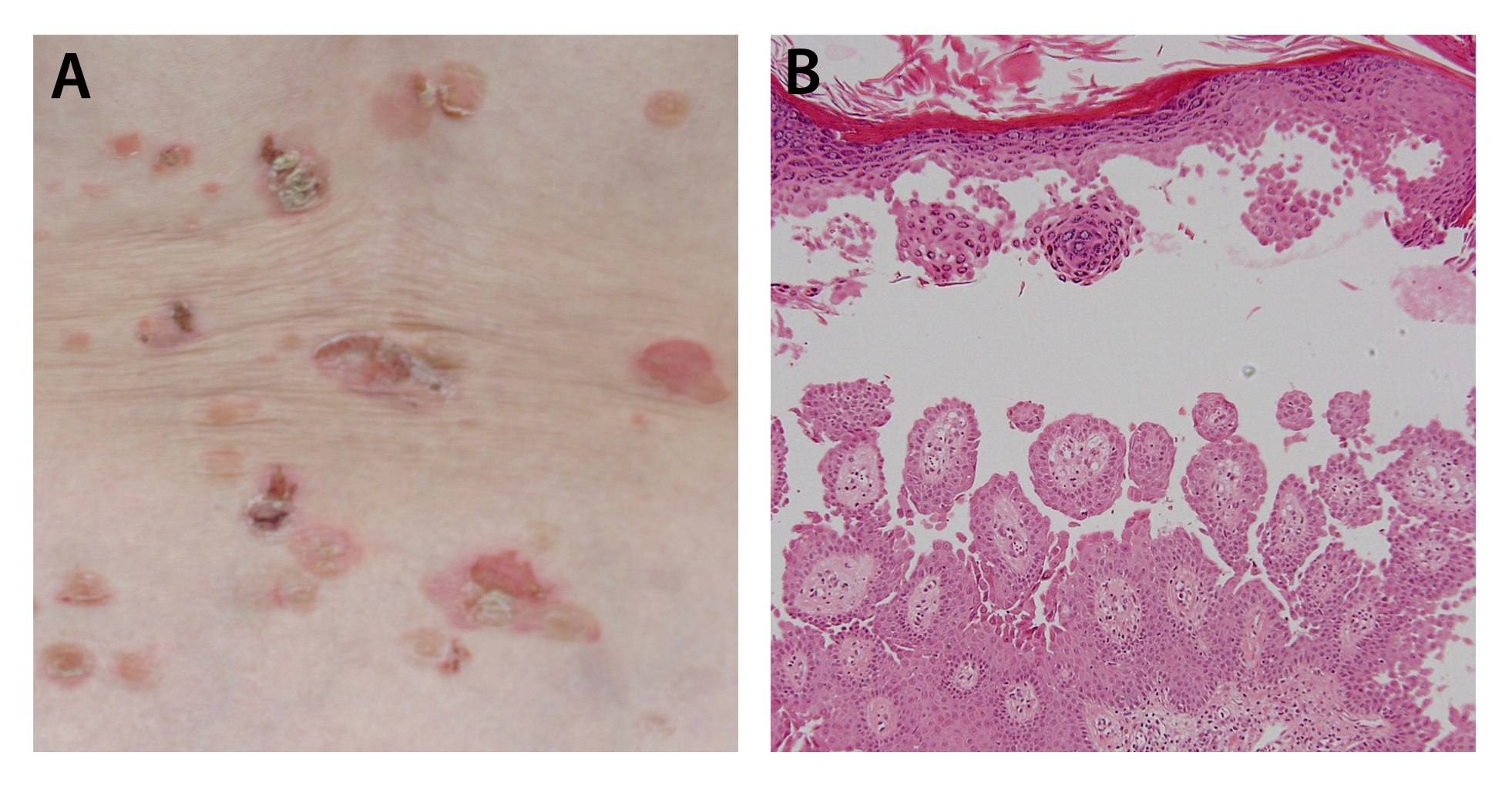

Supplement: Figure S4 — PV IgG directed against the Dsg3 EC1 domain are not required to cause blistering in vivo . Clinical presentation of lower back epidermal blisters (A) and histopathology (B) of patient PV IgG(b). Note that this patient lacks IgG directed against the Dsg3 EC1 domain (see Figure 7). (TIF) [file pone.0050696.s004.tif]

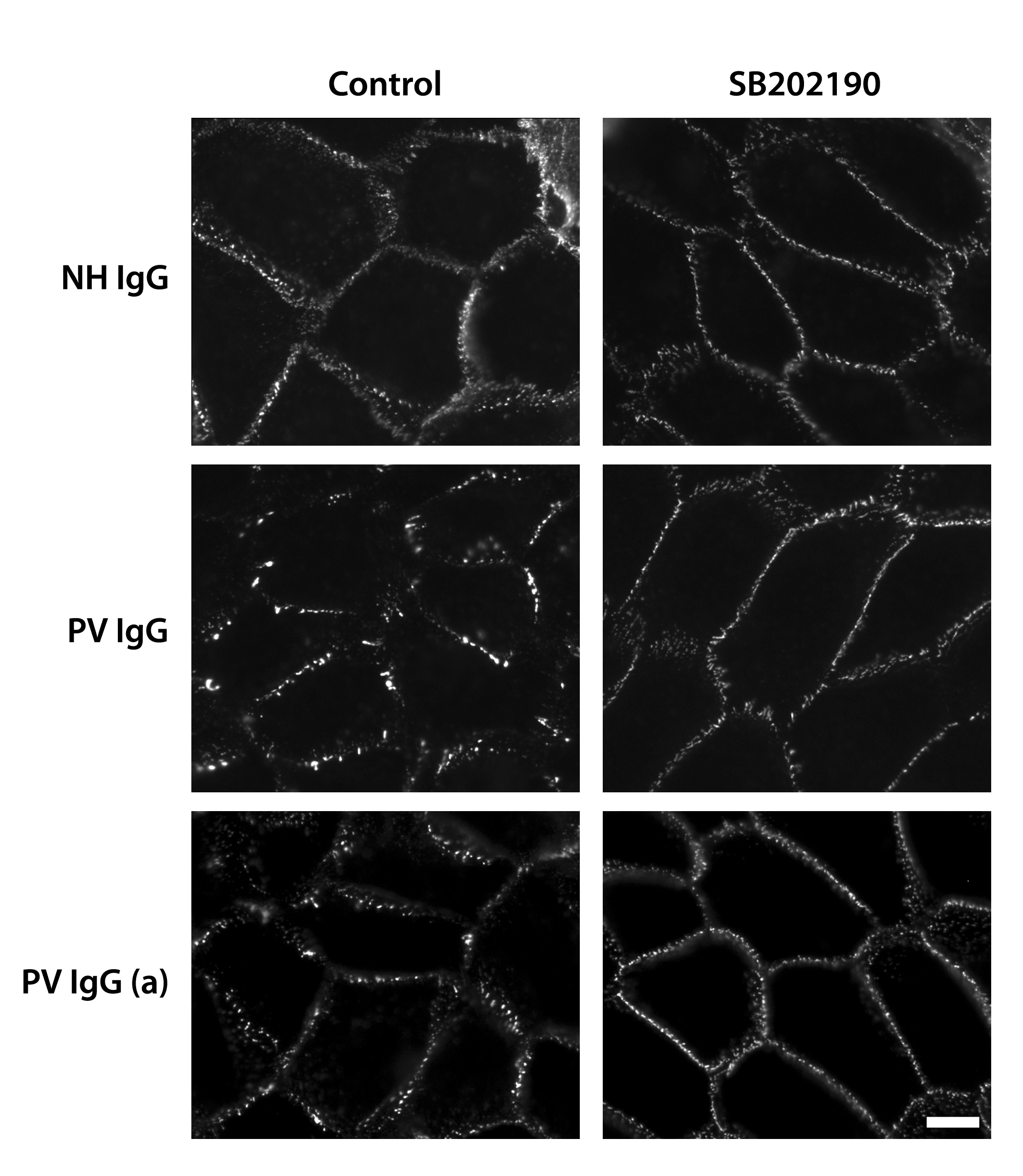

Supplement: Figure S5 — p38MAPK inhibition prevents Dsg3 clustering induced by PV IgG lacking EC1 antibodies. Cell surface Dsg3 was monitored using biotinylated AK23 followed by the addition of NH IgG, PV IgG and PV IgG (a). Cells were treated with the p38MAPK inhibitor SB202190 prior and during the addition of IgG. SB202190 prevented Dsg3 clustering induced by both PV IgG and PV IgG (a), the latter which only contains antibodies directed against domains EC3–4. (TIF) [file pone.0050696.s005.tif]
